# Combined miglustat and enzyme replacement therapy in two patients with type 1 Gaucher disease: two case reports

**DOI:** 10.1186/s13256-017-1541-7

**Published:** 2018-01-27

**Authors:** Dominick Amato, Mary Anne Patterson

**Affiliations:** 1 Mount Sinai Hospital, University of Toronto, Toronto, ON Canada; 2 Department of Medicine, Mount Sinai Hospital, 60 Murray Street, Room L-315, Box 34, Toronto, ON M5G 1X5 Canada

**Keywords:** Gaucher disease type 1 (GD1), Enzyme replacement therapy, Substrate reduction therapy, Combination treatment

## Abstract

**Background:**

Intravenous enzyme replacement therapy is a first-line therapy for Gaucher disease type 1, and substrate reduction therapy represents an oral treatment alternative. Both enzyme replacement therapy and substrate reduction therapy are generally used as monotherapies in Gaucher disease. However, one randomized study and several case reports have described combination therapy over short time periods.

**Case presentation:**

We report two female Gaucher disease type 1 patients of mainly Anglo-Saxon descent, where combined enzyme replacement therapy and miglustat substrate reduction therapy were administered to overcome refractory clinical symptoms. The first patient was diagnosed at age 17 and developed Gaucher disease-related bone manifestations that worsened despite starting imiglucerase enzyme replacement therapy. After switching to miglustat substrate reduction therapy, her bone symptoms improved, but she developed tremors and eventually switched back to enzyme replacement therapy. Miglustat was later recommenced in combination with ongoing enzyme replacement therapy due to continued bone pain, and her bone symptoms improved along with maintained visceral manifestations. Enzyme replacement therapy was subsequently tapered off and the patient has since been successfully maintained on miglustat. The second patient was diagnosed aged 3, and commenced imiglucerase enzyme replacement therapy aged 15. After 9 years on enzyme replacement therapy she switched to miglustat substrate reduction therapy and her core symptoms were maintained/stable for 3 years. Imiglucerase enzyme replacement therapy was later added as a boost to therapy and her symptoms were subsequently maintained over a 2.3-year period. However, miglustat was discontinued due to her relocation, necessitating an increase in enzyme replacement therapy dose. Overall, both patients benefited from combination therapy.

**Conclusion:**

While the majority of Gaucher disease type 1 patients will not need treatment with both substrate reduction therapy and enzyme replacement therapy, the current case reports demonstrate that judicious use of combination therapy may be of benefit in some cases.

## Background

Gaucher disease (GD) is a lysosomal storage disease caused by autosomal recessive mutations in the gene encoding acid beta-glucosidase (glucocerebrosidase; GBA). GD is one of the most common lipid storage diseases (LSDs), and has a worldwide incidence of between 1:40,000 and 1:86,000 [1–3]. Causal gene defects in GD lead to impaired intracellular lipid balance in cells of the monocyte/macrophage lineage, and subsequent infiltration of the liver, spleen, bone marrow and other tissues with lipid-laden macrophages. Core clinical manifestations include anemia, thrombocytopenia, hepatosplenomegaly, and skeletal disease [4–7].

Apart from bone marrow transplantation, which may be applicable only in rare situations, there are currently two approved treatment approaches for type I (non-neuronopathic) GD. Intravenous enzyme replacement therapy (ERT), comprising chronic biweekly infusions of recombinant analogs of human enzyme β-glucocerebrosidase, is considered as first-line therapy, and has been shown to improve hematologic parameters, organomegaly and, to a lesser degree, bone involvement [8–10]. Oral substrate reduction therapies (SRT) that target the enzyme, glucosylceramide synthase, represent an oral treatment alternative to intravenous ERT. Short-term clinical trials and long-term, open-label extension studies demonstrated the efficacy and safety of the first approved oral SRT, miglustat (Zavesca®, N-butyldeoxynojirimycin [NB-DNJ], Actelion Pharmaceuticals, Basel, Switzerland), in treating core hematologic and visceral symptoms of GD [11–13]. A long-term study has further shown that miglustat can be used as a maintenance therapy in patients with stable GD1 switched from previous ERT [14], and the effects of miglustat in improving bone pain and bone mineral density (BMD) over 2–12 years have been reported in other studies [15, 16].

While SRTs and ERTs are generally used as monotherapies in GD, one previous study has been reported in which combination therapy was used for a short time in GD1 patients [17]. There are also anecdotal reports of combinations of different agents being used in individual patients with neuronopathic GD (GD3) [18–20].

We report cases of two GD patients who received combined ERT and miglustat therapy to overcome refractory clinical symptoms: in the first case, intractable bone pain; and in the second, gastrointestinal symptoms and thrombocytopenia.

## Case presentation

### Patient 1 (see Fig. 1, left)

**Fig. 1:**
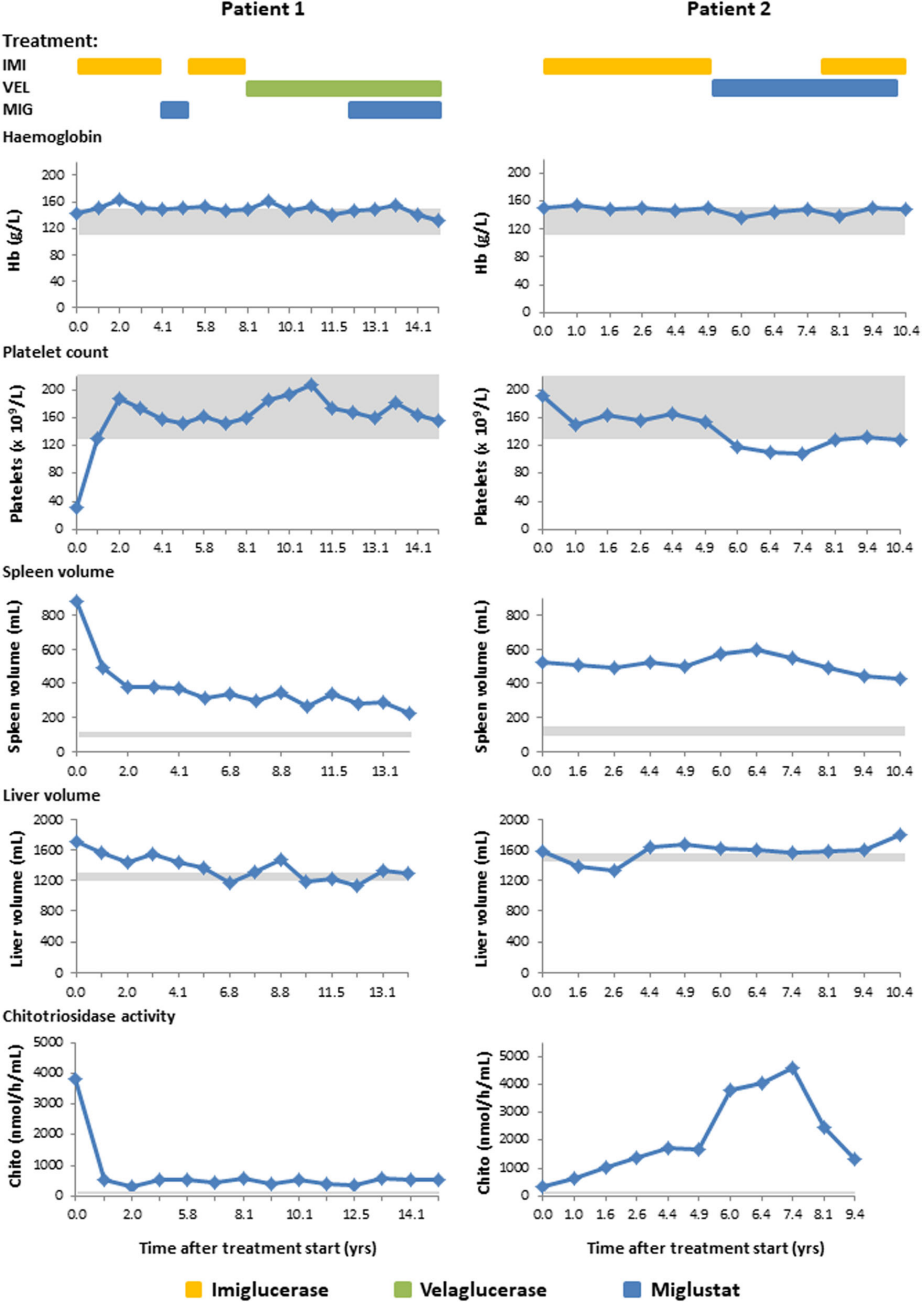
Core disease parameters relative to treatments in patients 1 and 2. *Colored bars* represent treatment timings in relation to x-axis time after treatment start. *Grey shaded areas* represent normal ranges for each parameter: Hb, 110–150 g/L; platelets 130–400 × 10^9^/L; chitotriosidase 4–120 nmol/hr/mL; spleen volume, (0.2% of body weight: patient 1 90–120 mL, patient 2 100–150 mL); liver volume (2.5% of body weight: patient 1 1200–1300 mL; patient 2 1450–1550 mL). *IMI* imiglucerase, *VEL* velaglucerase, *MIG* miglustat

This female patient was born in 1958 with Scottish, French-Canadian, and Micmac Indian heritage, and was diagnosed with GD at the age of 17, when she was found to have thrombocytopenia and splenomegaly. Her acid beta-glucosidase level was 2 nmol/mg protein/h (normal range: 8–16 nmol/mg protein/h) and genetic testing confirmed compound heterozygosity for the mutations N370S and H162P. She first presented to our clinic in 1986, aged 28 years, at which time her main complaints were easy bruising and left hip pain due to osteoarthritis. The only abnormal physical finding was palpable splenomegaly (spleen tip 2.5 cm below left costal margin). Her blood hemoglobin (Hb) and white cell count were normal but she had a low platelet count (68 × 10^9^/L). Bone marrow aspiration and biopsy showed Gaucher-cell infiltration with adequate megakaryocytes, suggesting splenic sequestration as the main cause of the thrombocytopenia. Imaging revealed avascular necrosis of her left hip.

Our patient was lost to follow-up for 16 years, and re-presented in 2002 with constant pain in her left hip as well as her mid and lower back. She still bruised easily, and complained of excessive fatigue. Although her Hb level and white cells remained normal she had severe thrombocytopenia (a platelet count of 28 × 10^9^/L) and very high plasma chitotriosidase activity [3840 (a marker of disease activity; normal range: 4–120 nmol/h/mL)] [21]. She was therefore started on biweekly infusions of imiglucerase ERT at 40 U/kg. Her hip and back pain continued to worsen, only partially relieved by strong analgesics and anti-inflammatories, and she underwent total left hip arthroplasty (avascular necrosis) in January 2005 after 3 years on ERT. While she showed initial improvement, she began having pains in various other bony areas requiring low-dose hydromorphone treatment. Her imiglucerase dose was increased to 60 U/kg every 2 weeks for 6 months, again without improvement.

In 2007, our patient switched to oral miglustat therapy after enrolling in clinical trial OGT-918-011, a long-term study of SRT maintenance treatment [14]. After 3 months’ treatment with miglustat 100 mg three times a day, her bone pains improved but she developed tremors. A reduction in the miglustat dose resulted in reduced tremors, but our patient’s bone pains recurred. She exited the trial after 3 months, and returned to imiglucerase at 40 U/kg every 2 weeks.

In 2009, our patient suffered a bone crisis, and in 2010 received four monthly doses of intravenous pamidronate, with no effect. Later that year, she switched from imiglucerase to velaglucerase at 60 U/kg every 2 weeks. This was eventually increased to 70 U, but with little effect on her chronic bone pains and narcotic analgesic use. From 2014 onward she took weekly alendronate, daily calcium, and vitamin D, but admitted to taking these medications erratically.

In 2014, miglustat (100 mg daily, gradually increasing to 100 mg three times a day) was added to the twice-weekly velaglucerase therapy. The doses of both medications were titrated to give maximal benefit with minimal side effects. Her bone pains improved appreciably on this combination. For the last 6 months of 2016, she was on miglustat 100 mg twice a day, with gradual reductions in velaglucerase dose; the latter was discontinued in December 2016. Since then she has been doing well on miglustat 100 mg twice a day, with no increase in bone pain, a gradual increase in stamina, and a decrease in fatigue. There have been no gastrointestinal side effects or any re-appearance of tremor.

Her plasma chitotriosidase level, apart from a brief spike to 1552 nmol/h/mL during the worldwide imiglucerase shortage, has consistently been in the range 300–600. Her bone marrow burden score [22] for the combined femora and lumbar spine has been 5 (out of 16) on two separate occasions. Magnetic resonance imaging of the lower extremities showed marrow replacement typical of GD and areas of infarction in the left femoral diaphysis. These findings have been unchanged since first recorded in 2002.

### Patient 2 (See Fig. 1, right)

The second female patient was born in 1983, and diagnosed in another country aged 3 years after presenting with marked hepatosplenomegaly. Her acid beta-glucosidase level was 1 nmol/mg protein/h, and she was a compound heterozygote for the N370S and RecNci1 mutations. After moving to Canada with her family aged 7 years, she was commenced on ERT with alglucerase aged 13 years. At age 15 years she switched to the recombinant imiglucerase.

In 2007, our patient enrolled in clinical trial OGT 918–011 with miglustat [14], and experienced intermittent side effects including mild tremor and an increase in bowel movements. She also lost approximately 10 kg body weight, and her platelet count dropped from 150–160 × 10^9^/L to 101 × 10^9^/L. She completed the trial in 2009 and continued on commercial miglustat. However, for the reasons mentioned above, in 2010 her miglustat dose was ‘boosted’ with imiglucerase ERT infusions, initially at a dose of 20 U/kg every 2 weeks. A prevailing worldwide shortage of imiglucerase forced a temporary drop to as low as 8 U/kg every 2 weeks, and the dose of miglustat was reduced from 100 mg three times a day to 100 mg twice a day. On this regimen, her gastrointestinal and neurological symptoms improved, she regained her original weight, and her platelet count increased to above 150 × 10^9^/L. Miglustat was discontinued in early 2013, because our patient and her husband moved to a different country, and the approval in Ontario to use combined therapy would not extend to another jurisdiction. The imiglucerase dose was increased to 27 U/kg every 2 weeks. During the 2.3-year period of combination therapy, her chitotriosidase levels fell from 4607 to 1339 nmol/h/mL, liver volume remained at 1.1 multiples of normal (MN), and spleen volume decreased from 4.9 to 3.7 MN.

This patient has not had any major bone problems. Magnetic resonance imaging has consistently shown the classic Erlenmeyer flask deformity as well as bone marrow infiltration, but there has been no change over the 14-year period from 2002 to 2016.

## Discussion

Miglustat has previously been shown to be an effective long-term maintenance treatment for core hematologic and visceral symptoms in GD [13, 16], and studies suggest that it can produce significant improvements in bone status, reducing bone pain as early as 6 months after treatment start [15]. In both cases presented here, miglustat administered as monotherapy demonstrated satisfactory maintenance of core GD disease parameters in patients stabilized on ERT, as has been reported previously [14]. However, patient 1 initially elected to stop miglustat due to a side effect commonly seen during initial therapy (tremor), and she recommenced miglustat several years later, at a gradually increasing dose, without a recurrence of tremor. Thus, the miglustat acted as a ‘booster’ to ERT in lessening bone pain in this patient [17, 18].

While patient 1 experienced rapid reductions in bone pain during her first treatment period on miglustat, other symptoms (principally tremor) prompted a return to ERT. However, her bone symptoms remained refractory to ERT, both with imiglucerase and later, with velaglucerase. This prompted the decision to try combination treatment, which resulted in significant improvements. In the several months since discontinuation of velaglucerase, our patient’s condition has remained stable.

Patient 2 remained on miglustat for more than 5 years, despite minor side effects of tremor and gastrointestinal disturbances. Core GD disease parameters were maintained during miglustat therapy after stabilization on ERT. We were unable to make further adjustments to her combination therapy after she moved to another country in 2013. In contrast to patient 1, in whom miglustat was added to ERT, in patient 2 ERT was added to miglustat (see Fig. 1). Interestingly, plasma chitotriosidase increased in patient 2 during the period of miglustat monotherapy, and decreased after the re-introduction of additional imiglucerase ERT. Similar findings were reported in a previous long-term study of miglustat therapy in patients stabilized on ERT [14].

There are few reports in the Gaucher literature regarding combination therapy with both ERT and SRT. In the only randomized trial to assess combination treatment, Elstein *et al.* reported data from 36 GD1 patients aged 17–69 years who had previously been stabilized on ERT, indicating that patients on combination treatment with miglustat plus ERT (imiglucerase) during a 6-month randomized phase showed significantly greater reductions in liver/spleen volume versus miglustat or imiglucerase monotherapy [17]. However, changes in hemoglobin concentration and platelet count were similar between monotherapy and combination therapy groups, and the number of patients was limited. Interestingly, during an open-label 18-month extension period where patients could continue on their randomized oral miglustat or intravenous imiglucerase monotherapies, or with combined SRT + ERT treatment, all patients chose to receive oral miglustat monotherapy. Ha *et al.* [23] reported the use of combined ERT and SRT in a GD1 patient to treat severe thrombocytopenia that had been refractory to ERT monotherapy. Although the SRT was eliglustat (Cerdelga®; Genzyme Corp, Cambridge, MA, USA), not miglustat, their experience lends weight to the notion that combination therapy, at least temporarily, may be of use in certain circumstances.

Other, anecdotal reports have addressed neuronopathic forms of GD and have focused on the effects on neurological symptoms that, while not relevant to GD1, pertain to the ability of miglustat to cross the blood–brain barrier [24]. Capablo *et al.* [18] described a marked improvement in neurological symptoms in a 31-year-old GD3 patient after 2 years on combined imiglucerase (60 U/kg every 2 weeks reduced from 240 U/kg dose) and miglustat (200 mg three times a day). In a case series of three pediatric GD3 siblings reported by Cox-Brinkman *et al.* [19] combined treatment with imiglucerase (30–120 U/kg every 2 weeks) and miglustat (dosed according to body surface area) resulted in stabilized neurological abnormalities in two patients. The third sibling, who received combination treatment from the age of 5 months, showed improvement/stabilization of neurological manifestations and normal psychomotor development. Most recently an Italian group treated a male Italian L444P homozygous infant with imiglucerase (60 U/kg every 2 weeks started at age 18 months) and miglustat (added at age 30 months) [20]. While the patient’s genotype predicted early-onset neurological manifestations, no neurological manifestations were observed throughout follow-up.

Overall, our two GD1 patients benefited from combination therapy, perhaps more in the case of patient 1 than in patient 2. Moreover, since discontinuation of ERT, patient 1 has continued to benefit from miglustat monotherapy.

## Conclusions

The great majority of GD1 patients will not need combination therapy with both SRT and ERT: the cost of either therapy alone is considerable. However, based on our experience in these two patients, we believe that judicious use of combination therapy may be of benefit in some cases.

## Abbreviations

BMD: Bone mineral density
ERT: Enzyme replacement therapy
GD1: Gaucher disease type 1
SRT: Substrate reduction therapy
